# An Atypical Presentation of Diffuse Basaloid Human Papillomavirus (HPV)-Related Squamous Cell Carcinoma of the Oral Cavity in the Setting of Prior Radiation

**DOI:** 10.7759/cureus.68780

**Published:** 2024-09-06

**Authors:** Jaswanthi Dogiparthi, Sidney Spencer, Jared Bunevich

**Affiliations:** 1 Medical Education, Lake Erie College of Osteopathic Medicine, Erie, USA; 2 Otolaryngology-Head and Neck Surgery, Mercy Health, Youngstown, USA

**Keywords:** atypical basaloid neoplasm, basaloid, basaloid variant of squamous cell carcinoma, head and neck neoplasms, hpv-related hnscc, hpv-related oropharyngeal cancer, oral cancers

## Abstract

Basaloid squamous cell carcinoma (BSCC) is a rare subtype of squamous cell carcinoma (SCC) that can occur in the head and neck region. This particularly aggressive type of SCC has been linked to human papillomavirus (HPV) and carries a better prognosis when found in the oropharynx. We present a rare manifestation of oropharyngeal basaloid HPV-related SCC in a 75-year-old female with a history of prior radiation to the head and neck area for moderately differentiated SCC of the epiglottis. The patient presented with an erythematous rash-like mucosal lesion that extended from the oral vestibule and mucosa of the lower lip to the right buccal trigone, without any mass lesions. The case presented here is unique due to the presence of oral HPV-related BSCC in the setting of a past medical history of prior head and neck radiation. The nature of this lesion can result in late-stage diagnosis and poor patient outcomes. The uncharacteristic presentation seen in this patient emphasizes the importance of early diagnosis and management. Awareness of a variety of presentations of this aggressive cancer type is warranted due to the poor prognosis this variant carries, especially when diagnosed in advanced stages.

## Introduction

Basaloid squamous cell carcinoma (BSCC) is an aggressive variant of squamous cell carcinoma (SCC) often seen in the head and neck region, particularly the base of the tongue, hypopharynx, and larynx, and less frequently in the mouth and oral mucosa [1,2]. First described in 1986 by Wain et al., BSCC is a high-grade and aggressive tumor with histopathological features of basal and squamous cell components and an increased propensity for metastasis especially to the lungs and liver [3,4]. The lesion has been previously described as capable of recurrence, deep invasion, and lymph node involvement [1,3]. Although the etiology of BSCC and conventional oral squamous cell carcinoma (OSCC) are similar, such as the history of smoking and alcohol exposure, recent studies have seen increased occurrence of BSCC with human papillomavirus (HPV) found in the basaloid tumor, particularly of HPV genotype 16 seen in younger non-smoking patients [3]. Here, we report a unique case of diffuse, multifocal HPV-related BSCC in a 75-year-old female with a previous history of radiation therapy for SCC of the epiglottis. 

## Case presentation

**Figure 1:**
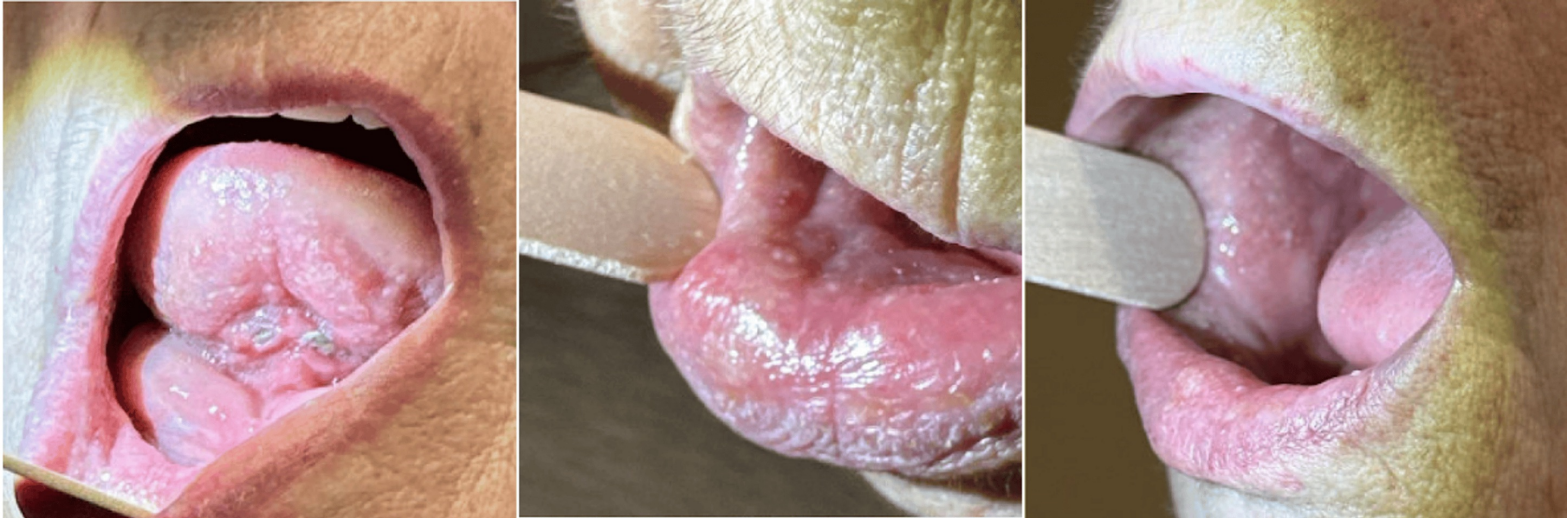
Images of the lesions in the oral cavity The initial oral cavity exam demonstrated a diffuse, white, papular rash to the right buccal and lower lip mucosa.

The patient is a 75-year-old female with a prior history of chemotherapy and radiation for invasive, moderately differentiated SCC of the epiglottis (T2N2a) in 2012. She presented originally with symptoms of velopharyngeal insufficiency with nasopharyngeal regurgitation in the summer of 2023 but was later worked up for oral lesions. The patient had dental implants placed in the mouth in March and underwent 40 hyperbaric oxygen treatments prior to this. She has a history of heavy drinking and a 30-pack-year smoking history. Physical exam at that time demonstrated an elevated, erythematous rash-like mucosal lesion that extended from the oral vestibule and mucosa of the lower lip to the right buccal trigone area without significant induration. After a two-week trial of Decadron swish and spit did not resolve the lesions, a biopsy was taken from the lesions on the lower lip mucosa in the office as seen in Figure 1. The pathology returned as carcinoma with squamous features involving the minor salivary gland structures. The patient was subsequently taken to the operating room for further biopsies.

**Figure 2:**
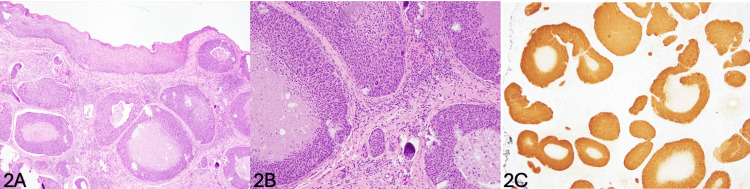
Pathology slides from oral cavity biopsies (2A) Low-power magnification (4×, H&E stain) normal squamous epithelium with tumor nests in the subcutaneous tissue. (2B) Higher-power magnification (10×) tumor includes lymphovascular invasion and central necrosis. (2C) p16 HPV marker immunostain is diffusely and strongly positive. HPV: human papillomavirus

Biopsies from the right lower lip and right buccal mucosa in Figure 2A-2B demonstrate sections of variably sized nests of basaloid neoplastic epithelioid cells in the subepithelial soft tissue with some of the larger ones showing central, comedo-like necrosis and some smaller ones appeared to be present in the lymphatic channels. The final pathology returned as HPV-related BSCC diffusely and strongly positive for p16, a marker for HPV, as seen in Figure 2C. There was no direct connection between the tumor nests and the surface squamous epithelium, and the surface squamous epithelium showed no evidence of dysplasia or carcinoma in situ. PET scan demonstrated areas of intense activity within the neck including the left parapharyngeal space and in anterior to the left carotid sheath concerning metastatic disease as well as activity along the hyoid. The patient was referred to a pulmonologist for associated findings of a lung nodule with potential for malignancy and lymphadenopathy and underwent endobronchial ultrasound, broncho-alveolar lavage, and trans-bronchial needle aspiration of the concerning lymph node. All cytology was negative for malignant cells. Given the patient had positive biopsies for BSCC at two separate sites located centimeters apart, the decision was made to seek a separate opinion from the university system. The patient was deemed a non-surgical candidate due to extensive and diffuse involvement and the debilitation that radical resection would cause. Additionally, the patient had significant sequelae from her prior treatment from SCC of the epiglottis. After a multidisciplinary tumor board, the recommendation was for systemic therapy and radiation. The patient underwent a total of 28 fractions of radiation to the anterior oral cavity and zone IA/B with a boost to the anterior oral cavity of 12 fractions and received a total dose of 74 Gy for two months. The patient also received eight cycles of cisplatin. A post-treatment three-month PET scan demonstrated at least partial treatment response with significant improvement of the metabolically active disease in the left neck and along the hyoid. There were some residual areas noted with increased activity. Of note, the patient did present to the ED six months post-treatment with significant inspiratory stridor and respiratory distress and ultimately underwent awake tracheostomy due to a non-functional larynx.

## Discussion

Although the etiology and risk factors for BSCC and conventional OSCC are similar, such as a history of smoking and alcohol exposure, increasing evidence of an association between viral DNA in BSCC specimens and incidence has been established. One study by Kleist et al. found a statistically significant detection rate for HPV DNA of 32.5% in BSCC and for HSV DNA of 6.5% with and without co-infection with HPV [5]. The significant correlation between HPV and basaloid morphology of SCC makes a viral carcinogenic association increasingly prevalent and worth considering from a clinical standpoint. HPV-related BSCC with p16-positive immunohistochemistry has also been identified to be an indicator for a better prognosis when in the setting of oropharyngeal cancer [6]. A study done by Cooper et al. found a five-year disease-specific survival of 73% for p16-positive tumors compared to 35% for p16-negative tumors and 74% for basaloid differentiated tumors compared to 41% for non-basaloid differentiated tumors [6]. This study also suggested that basaloid differentiated tumors and p16 positivity in oropharyngeal cancer may correlate directly. Although p16 carries an association for better prognosis, this highly aggressive and invasive variant of OSCC is often detected at an advanced stage due to the possibility of subtle signs and symptoms experienced by the patient and therefore carries an overall poor prognosis. 

Due to the high likelihood of metastasis, as seen in this patient, treatment typically involves radiation and chemotherapy. However, the combined use of systemic and intratumoral administration of the HPV vaccine to treat BSCC was first introduced and described by Nichols et al. in 2018 [7]. This case described the use of a nine-valent HPV vaccine therapeutically for cutaneous BSCC which resulted in the regression of tumors in a 90-year-old female, suggesting a new approach to treatment when surgery may not be an option, such as the patient described in this case. Further investigation regarding the use of a nine-valent HPV vaccine for oral BSCC may suggest using the vaccine as a novel potential treatment option. 

Additionally, the case presented is unique in its presentation of HPV-related BSCC recorded in the setting of a past medical history of prior head and neck radiation. Radiation is a known risk factor of cancer and adds to this patient's risk factors along with a history of a prior primary cancer and excessive smoking and alcohol consumption. With the rising incidence of HPV-related BSCC, it is important to consider the diagnosis, particularly in those with significant risk factors. Additionally, this patient presented with an erythematous rash-like lesion with no obvious mass, making the diagnosis less apparent and delaying treatment. This case introduces the consideration of radiation history in the initial differential diagnosis of HPV-related BSCC. Given that this diagnosis may present in advanced stages, an increased degree of suspicion may aid in timely diagnosis and treatment, allowing for better prognosis and management. 

## Conclusions

While HPV-associated oropharyngeal BSCC portends a more typical presentation and improved prognosis, HPV-associated BSCC is much far less common and has a very poor prognosis. In this case, the cancer was detected in a mucosal rash in the setting of prior radiation. It is essential to biopsy any suspicious lesion as cancer can present atypically such as was the case in this patient without any obvious mass lesion.
